# Compatibility Evaluation of Clustering Algorithms for Contemporary Extracellular Neural Spike Sorting

**DOI:** 10.3389/fnsys.2020.00034

**Published:** 2020-06-30

**Authors:** Rakesh Veerabhadrappa, Masood Ul Hassan, James Zhang, Asim Bhatti

**Affiliations:** Institute for Intelligent Systems Research and Innovation, Deakin University, Melbourne, VIC, Australia

**Keywords:** extracellular, micro-electrode array, spike sorting, clustering, validation indices

## Abstract

Deciphering useful information from electrophysiological data recorded from the brain, *in-vivo* or *in-vitro*, is dependent on the capability to analyse spike patterns efficiently and accurately. The spike analysis mechanisms are heavily reliant on the clustering algorithms that enable separation of spike trends based on their spatio-temporal behaviors. Literature review report several clustering algorithms over decades focused on different applications. Although spike analysis algorithms employ only a small subset of clustering algorithms, however, not much work has been reported on the compliance and suitability of such clustering algorithms for spike analysis. In our study, we have attempted to comment on the suitability of available clustering algorithms and performance capacity when exposed to spike analysis. In this regard, the study reports a compatibility evaluation on algorithms previously employed in spike sorting as well as the algorithms yet to be investigated for application in sorting neural spikes. The performance of the algorithms is compared in terms of their accuracy, confusion matrix and accepted validation indices. Three data sets comprising of easy, difficult, and real spike similarity with known ground-truth are chosen for assessment, ensuring a uniform testbed. The procedure also employs two feature-sets, principal component analysis and wavelets. The report also presents a statistical score scheme to evaluate the performance individually and overall. The open nature of the data sets, the clustering algorithms and the evaluation criteria make the proposed evaluation framework widely accessible to the research community. We believe that the study presents a reference guide for emerging neuroscientists to select the most suitable algorithms for their spike analysis requirements.

## 1. Introduction

**Figure 1:**
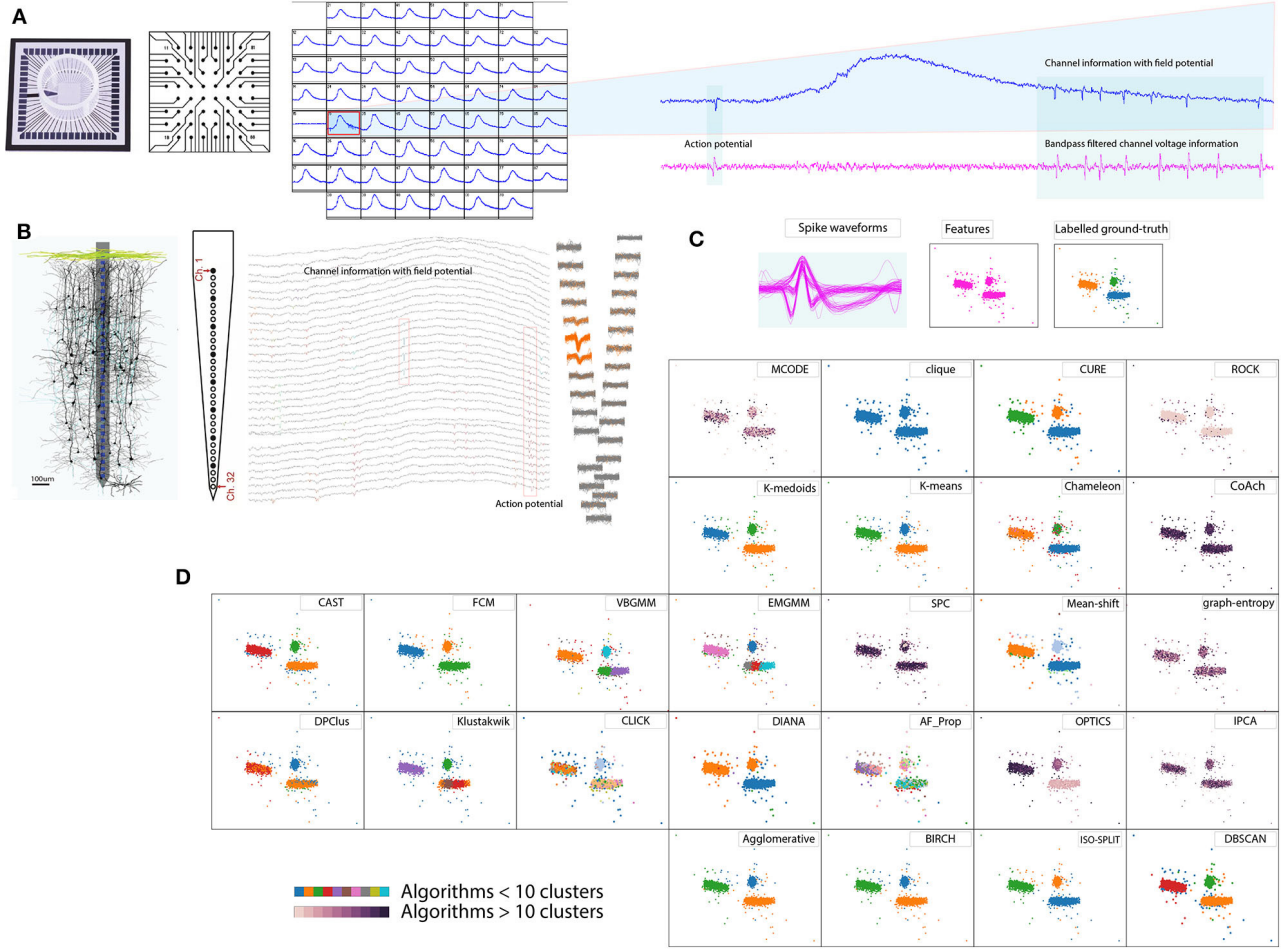
Spike sorting overview. **(A)** *In vitro* method 60 electrode MEA, schematic cross-section and example recordings (Gaburro et al., 2018a,b). **(B)** *In vivo* method 32 electrode array, schematic cross-section and example recordings (Schjetnan and Luczak, 2011; Rossant et al., 2016). **(C)** Pool of spikes extracted from the recordings, features extracted, and labeled *ground-truth* (Quiroga et al., 2004). **(D)** Example of cluster algorithms results addressed in the report for the data set in **(C)**, demonstrating diverse choices available for spike sorting.

Recording electrophysiological activity of neuronal circuits is in practice for over a century and has been facilitating numerous research interests (Hong and Lieber, 2019). An aggregate of electrophysiological technique is Extracellular method employing electrodes to study neural activity. The recordings can be acquired through *in-vitro* (non-invasive) or *in-vivo* (invasive) methods. In early extracellular studies, neurophysiologists demonstrated the electrical activity in larger neurons and axons (for example employed giant axons of width 0.5–1 mm dissected from squids) using electrodes of diameter 100 μ*m* and length 10–20 mm. However, a refined method was formally demonstrated by Hubel in 1957 using electrodes with much finer tips (1–10 μm diameter) which could record neural activity from relatively smaller nerves and axons (Hubel, 1957). Figure 1A shows *in-vitro* methods which are in practice especially in pharmacological studies (Jenkinson et al., 2017; Mulder et al., 2018), investigation of neurotropic viral activity in mammalian and mosquito brain (Gaburro et al., 2018a,b) or development of cures to neurological dysfunctions by studying the behavior of collective network activity (Md Ahsan Ul Bari and A., 2017; Gamble et al., 2018). Similarly, Figure 1B shows *in-vivo* methods which are prevalent in developing remedies for many neurological dysfunctions, including treatment of paralysis through stimulation (Assad et al., 2014; Liu et al., 2017).

Raw extracellular recordings comprise of low frequency component (100–300 Hz, Quiroga et al., 2004) reflecting transmembrane activity from a population of neurons referred to as local field potential Figures 1A,B and short lived component (3–3.2 ms, Prentice et al., 2011; Veerabhadrappa et al., 2017) reflecting activity from a neuron or single units referred to as action potentials Figures 1A,B or spikes (Gold et al., 2006). To facilitate further analyses related to deciphering activities in the brain, extracting and sorting of spikes referred to as *spike sorting* is an essential process. Human operators were used to manually sort the spikes by identifying the distinct shape of action potentials (Meister et al., 1994) whereas some procedures used a combination of clustering and human operators (Rossant et al., 2016).

Advancements in neurophysiological research saw introduction of multiple electrodes (tetrode) in 1983 (McNaughton et al., 1983; Hong and Lieber, 2019) for *in-vivo* recording and array of microelectrodes (Meister et al., 1994) for *in-vitro* studies. Meister et al. (1994) demonstrated the use of microelectrode array comprising of 60 electrodes to study retinal ganglion cells. Over the last three decades, neurophysiological studies have seen a significant rise in development and application of dense microelectrodes (Hong and Lieber, 2019). The order of simultaneous recording channels on dense electrode arrays ranges from 32, 60, 512, 4,096 up to 11,000 (Rossant et al., 2016; Jäckel et al., 2017; Gaburro et al., 2018a; Hong and Lieber, 2019). As a consequence of dense electrode array and longer recording duration, the data generated by systems are fairly large, which could range from several giga-bytes to tera-bytes, referred to as big-data in present trends. Such neuronal recordings are often processed off-line using spike sorting algorithms. Figure 1 shows an overview of the spike sorting algorithm. In general, the spike sorting procedures (Lewicki, 1998; Quiroga et al., 2004; Prentice et al., 2011; Ekanadham et al., 2014; Rey et al., 2015; Niediek et al., 2016; Rossant et al., 2016; Veerabhadrappa et al., 2017) involve pre-filtering of raw voltage signals to remove field potential (Figures 1A,B), detection and extraction of spike events using standard thresholding techniques (Figure 1C), representation of spike waveforms by features, such as wavelets (Quiroga et al., 2004) or principal component analysis (PCA) (Rossant et al., 2016), and identification of unique clusters (Figure 1D).

The results of clustering are extremely important for deriving statistical analyses; inter-spike intervals (Li et al., 2018), correlogram analysis (Harris et al., 2000), spike rates (Pillow et al., 2013; Ekanadham et al., 2014; Veerabhadrappa et al., 2016), and detection of bursting neurons (Lewicki, 1998; Rey et al., 2015). Value of knowledge disseminated from many experimental studies depends on the accuracy of results obtained by spike sorting algorithms. For example, probabilities derived from spike rates are employed in the identification of spike classes contributing to overlapping spike events (Pillow et al., 2013; Ekanadham et al., 2014; Veerabhadrappa et al., 2016). The spike sorting procedures strongly depend on clustering algorithms as a primary approach to distinguish spikes with minimal or no human intervention (Pachitariu et al., 2016; Rossant et al., 2016). The lack of accepted protocol has seen several dissimilarities between clusters formed by human operators (Gray et al., 1995; Harris et al., 2000; Rossant et al., 2016). In a previous study (Shan et al., 2017), it is very well-established that the quality of initial estimations will determine efforts required by human operators to isolate the clusters satisfactorily.

Many clustering algorithms have been debated, evaluated and compared as the best choice for automatic spike sorting. Researchers agree that to date there is no accepted clustering procedure to sort spikes (Pillow et al., 2013; Ekanadham et al., 2014; Rey et al., 2015). It has also been argued whether there is a need for spike sorting algorithms and how future extracellular processing could be shaped (Rey et al., 2015). The disagreement between procedures could be arising from volatility of data being investigated, recording equipment, background noise, the activity of culture (*in-vitro*) (Shoham et al., 2003; Choi et al., 2006; Paralikar et al., 2009; Pillow et al., 2013; Takekawa et al., 2014). Further, owing to the nature of large data sets, dependency on the robustness of clustering algorithms and feature selection process has increased (Harris et al., 2000; Quiroga et al., 2004; Prentice et al., 2011; Ekanadham et al., 2014; Rossant et al., 2016; Veerabhadrappa et al., 2017).

As opposed to questioning the necessity of spike sorting, considering the above facts, it is in our best interest to address the challenge via a contemporary approach. One similar approach was discussed by Shan et al. (2017) where procedures were customized depending on the brain region under investigation. The idea of our study is to cover an essential portion of the groundwork that can assist in contemporary spike sorting procedures. Figure 1D demonstrates an example of the diverse nature of algorithms where some algorithms returned less than 10 clusters, while some returned over 10, indicating that no two results of clustering algorithms match. Among the algorithms exceeding 10 clusters, ordering points to identify clustering structure (OPTICS) returned the lowest clusters equivalent to 24. In a worst-case graph_entropy generated clusters over 150. Thus evaluation is necessary to determine choices of algorithms available for spike sorting. Results presented in the report helps researchers involved in the processing of extracellular data to select an appropriate clustering algorithm. Some review articles have criticized that when a new algorithm is introduced, the results are often biased and *ad-hoc* quantitative metrics are used to compare performances between peer algorithms or its predecessor (Amancio et al., 2014). The quality of a clustering algorithm should instead be evaluated using accuracy (Amancio et al., 2014).

**Figure 2:**
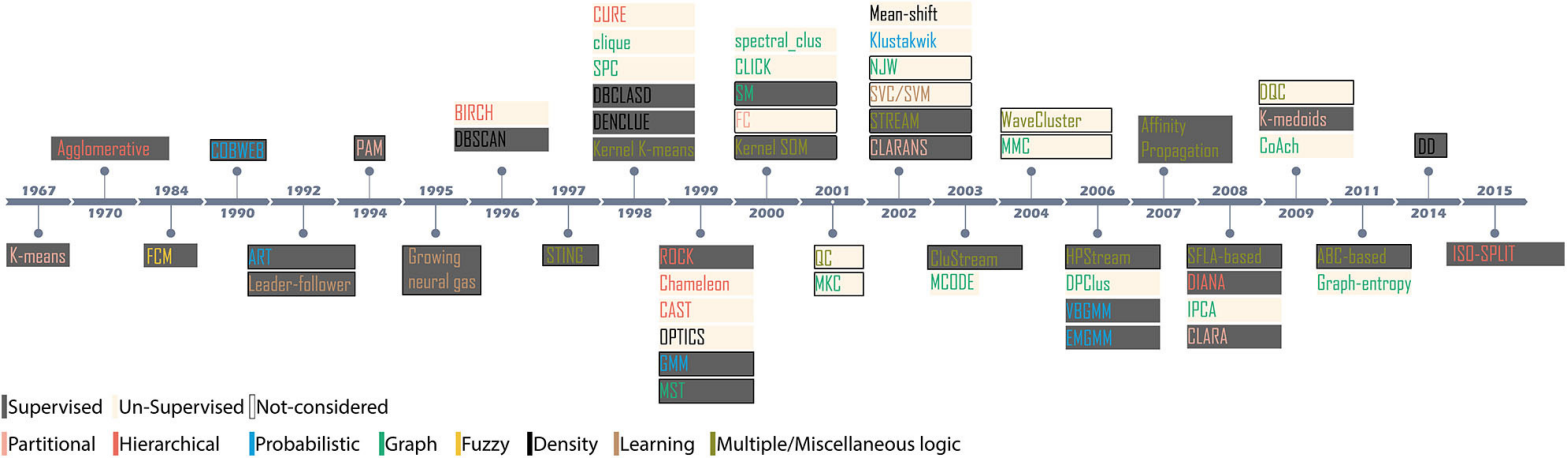
Evolution of novel clustering algorithms since 1967. The shaded region indicates the methodology; supervised or unsupervised for the clusters considered in the context, and the colored text indicates the category of the clustering algorithm.

There is no preconceived hypothesis to accept or reject a clustering outcome. Instead, cluster results must be accepted through exploratory methods (Kaufman and Rousseeuw, 2008b). The current study aims to present completely unbiased evaluations. All materials such as data sets, clustering algorithms, feature extraction processes and evaluation criteria employed in this study are open source. Supplementary resource presented in Appendix C lists all the algorithms used in our study and accessible for cross-verification. We assessed the performance of 27 algorithms (Figure 2) which covers almost all of the underlying clustering theories. The algorithms are paired with two most widely adopted feature extraction methods (Rey et al., 2015); PCA (Harris et al., 2000; Shoham et al., 2003; Pachitariu et al., 2016; Rossant et al., 2016) and wavelet decomposition of spike waveforms (Hulata et al., 2002; Quiroga et al., 2004; Takekawa et al., 2010; Niediek et al., 2016). As a standardized approach, we employ the data sets made available by Quiroga et al. (2004). The ground-truth associated with the data sets (Harris et al., 2016) provides information about the noise level, number of channels, number of spikes, number of overlapping spikes, number of spike waveform classes and time of spike origin thereby, establishing a standard platform to assess the results of clustering algorithms.

Our study employs internal and external validation indices to evaluate the performance of cluster algorithms. Here, internal indices satisfy quantitative criteria (Zhang et al., 2018) and external indices satisfies qualitative criteria (Hullermeier et al., 2011) addressing evaluation concerns raised in previous reviews (Amancio et al., 2014). Furthermore, to complement the results of indices we employed two additional qualitative measures accuracy and confusion matrices which are alternative to some popular methods such as false negatives and false positives, previously employed in the evaluation of spike sorting algorithm (Harris et al., 2000). The results and analysis are presented in terms of supervised and unsupervised approach, consistency of an algorithm across different sets of data and features.

The experiments conducted in this study demonstrates the competency of clustering algorithm across six feature-sets. We have explored how indices vary across different feature-sets and their impact on the decision of identifying good clusters. The evaluation comprising of qualitative and quantitative aspects provide readers with a comprehensive understanding of algorithms used in the study. Although the results presented in our report uses extracellular data, it will also benefit people across various research communities. The technique offers a different dimension to evaluate the performance of clustering algorithms where results from all algorithm-features pair are compared during analysis. For ease of following acronyms in the article, a list of abbreviations is provided in Appendix A.

## 2. Evolution of Clustering Algorithms

Since 1967, it is recorded that more than 60 novel and numerous *ad-hoc* clustering algorithms have been introduced for applications in myriad domains of data analysis. However, relatively few algorithms have been debated across spike-sorting community (Shoham et al., 2003; Rey et al., 2015). Figure 2 highlights the evolution of clustering since the introduction of K-means in 1967. Based on the methodology, the algorithms can be either supervised or unsupervised. A supervised clustering requires users to perceive prior knowledge about the possible partitions. For example, K-means requires users to specify the partition level (Xu and Wunsch, 2009). Appropriate cluster partition can be selected either by visually observing the feature space or, evaluating validation score using internal indices (Kaufman and Rousseeuw, 2008b; Buccino et al., 2018). For example, the clusters generated by the mixture of Gaussians algorithm is evaluated using *calinski*−*harabaz* before an appropriate cluster is considered (Buccino et al., 2018). Unsupervised algorithms are pre-set with decision-making parameters. For example, the algorithm superparamagnetic clustering (SPC) (Blatt et al., 1996) is pre-set with the nearest neighbor distances, the number of Monte-Carlo spins and the cluster decision is made by evaluating paramagnetism using a pre-set range of temperature (Quiroga et al., 2004). Algorithms also employ internal indices to evaluate the best clustering outcome. For example, Klustakwik automatically evaluates the clusters using best ellipsoidal error rate while an in-built cost function checks for best score (Rossant et al., 2016).

### 2.1. Cluster Groups

Clustering algorithms can loosely be categorized into seven groups based on the underlying principle concepts. Since their introduction, several algorithms evolved through enhancement and augmentation of new features catering to emerging needs in data analysis. Clustering algorithms are often revised to fit better to a problem being investigated and later generalized to be applicable for another domain. For simplicity in understanding, we broadly classify the algorithms as Partitional, Hierarchical, Probabilistic, Graph-theory, Fuzzy logic, Density-based, and Learning-based (Xu and Wunsch, 2009; Xu and Tian, 2015).

#### 2.1.1. Partitional

Partitional clustering tends to sub-divide the entire feature vectors to form clusters based on the density of points around median or centroid. One of the early uses of partitional based clustering in spike sorting was K-means, introduced by Salganicoff et al. (1988) in 1988. K-means and K-medoids are two algorithms considered in our study. Other popular algorithms from the same family are partitioning around medoids (PAM), Clustering Large Applications (CLARA), and Clustering Large Applications based on Randomized Search (CLARANS) (Kaufman and Rousseeuw, 2008c). K-means has also been at the center of two recently published algorithms (Pachitariu et al., 2016; Caro-Martín et al., 2018).

#### 2.1.2. Hierarchical

Hierarchical clustering algorithms use a dendrogram or binary tree structure based on the separation between points. The tree runs from highest order through to successively sub-dividing the feature points until all N points in the feature-set are completely isolated. All the techniques presented in the report uses Euclidean distance for establishing the separation between points. An appropriate subdivision level of the tree representing clusters is dependent on the underlying supervised or unsupervised nature of the algorithm. Fee et al. (1996) in 1996 introduced the hierarchical clustering algorithm to sort spikes. The evaluation presented in report considers ISO-SPLIT Magland and Barnett (2015) [considered for Mountain sort Chung et al. (2017)], Cluster Affinity Search Technique (CAST) Ben-Dor et al. (1999); Howe et al. (2010) Divisive Analysis (DIANA) Kaufman and Rousseeuw (2008a), clustering using representatives (CURE) Guha et al. (1998), Chameleon Karypis et al. (1999), Agglomerative, robust clustering (ROCK) Guha et al. (1999); Novikov (2019) and Balanced Iterative Reducing and Clustering using Hierarchies (BIRCH) Zhang et al. (1996) for evaluation.

#### 2.1.3. Probabilistic

Probabilistic clustering algorithms are prevalent in spike sorting. The rationale tends to maximize the posterior distribution based on the likelihood of a data-point/vector belonging to a class, thereby targeting the spike sorting as a non-parametric problem. Several combinations based on Expectation Maximization (EM) (Dempster et al., 1977; McLachlan and Krishnan, 2007), variational bayesian (Attias, 2013) and Multivariate t-distribution (Shoham et al., 2003) (also referred to as t-distribution) with mixture model (Law et al., 2004) or Gaussian mixture models (GMM) (Jin et al., 2005) have been explored in the past. Lewicki's Lewicki (1994) model introduced in 1994 incorporates iterative approach on the theory of maximum likelihood of a spike waveform being an instance of a class. Further, the bayesian-based clustering algorithm was introduced for template matching in 1998 (Lewicki, 1998). Harris et al. (2000) employed Chi-Square distributions to distinguish spike waveforms. A model based on multivariate T-distributions was introduced by Shoham et al. (2003) and Shan et al. (2017). GMM based approach was introduced to spike sorting by Sahani et al. (1998). Takekawa and Fukai (2009) in his study explored variational bayesian inference gaussian mixture model (VBGMM) and expectation maximization based gaussian mixture model (EMGMM) based clustering algorithm.

Clustering algorithm based on EM was also explored through Klustakwik. Records suggests the existence of Klustakwik, since 2002, as part of MClust toolbox (Wild et al., 2012) and template-matching version mentioned by Blanche et al. (2004), an unsupervised version was documented by Kadir et al. (2014). The klusta suite with graphical user interface and other statistical functionality is documented by Rossant et al. (2016). GMM with Kalman filter employing probabilistic learning and hidden markov model was documented by Calabrese and Paninski (2011). Dirichlet process was explored by Gasthaus et al. (2009). Mixture Model, which uses dirichlet process to iteratively identify distribution in the data and forms clusters, was documented by Carlson et al. (2013). The evaluation presented in report considers Klustakwik, VBGMM (Bishop, 2006) and EMGMM (Bishop, 2006) for evaluation.

#### 2.1.4. Graph-Based

The Graph-based algorithms are also equally popular in bio-medical signal processing, especially clustering cancer data (Howe et al., 2010), gene expression classification (Sharan and Shamir, 2000) or protein label classification (Kenley and Cho, 2011), to mention a few. Wave_clus (Quiroga et al., 2004) and Combinato Niediek et al. (2016) built around SPC (Blatt et al., 1996), follows a similar technique incorporating Minimal Spanning Tree (MST) and K-nearest neighbour (KNN) Wave_clus is a very popular algorithm and its graphical user interface provides comprehensive details of spike sorting process. The evaluation presented in report considers Cluster Identificaton using Connectivity Kernels (CLICK) (Sharan and Shamir, 2000; Shamir et al., 2005), clique (Palla et al., 2005; Price et al., 2013), divisive projected clustering (DPClus) (Altaf-Ul-Amin et al., 2006; Price et al., 2013), graph-entropy (Kenley and Cho, 2011; Price et al., 2013), core-attachment method clustering (CoAch) (Wu et al., 2009; Price et al., 2013), influence power based clustering algorithm (IPCA) (Li et al., 2008; Price et al., 2013), spectral clustering (Shi and Malik, 2000; Pedregosa et al., 2011), and molecular complex detection (MCODE) (Bader and Hogue, 2003; Price et al., 2013).

#### 2.1.5. Fuzzy Logic

Fuzzy C-Means (FCM) is one of the popular clustering employing Fuzzy logic. The logic of FCM derives from the concept of both partitional and probabilistic clustering. Partitions are formed by constantly reducing the cost function. The fuzzy theory was introduced into spike sorting by Zouridakis and Tam (2000). The evaluation presented in the report considers just FCM for evaluation.

#### 2.1.6. Density-Based

As the name suggests, the theory is constructed around the density of data points, and the cluster shape is not a confined factor. Density Based Clustering of Applications with Noise (DBSCAN) is a popular clustering algorithm in this family. The clustering result is decided by two parameters; a minimum number of points *Minpts* that must be included in a cluster and *Eps*, the epsilon value which specifies radius to form clusters. Knowledge of *Minpts* and *Eps* is arbitrary (the best outcome is performed through several trials by varying the parameters) and hence makes the process supervised. A similar clustering algorithm OPTICS was employed in spike sorting by Prentice et al. Prentice et al. (2011). Early use of Mean-shift Fukunaga and Hostetler (1975) clustering algorithm in spike sorting was introduced by Swindale and Spacek (2014), in the form of gradient ascent clustering. The evaluation presented in report considers OPTICS, DBSCAN, and Mean-shift.

#### 2.1.7. Learning-Based Clustering

Learning-based algorithms such as Self-organizing Maps require prior understanding or initial ground-truth in the form of training sets to train their weights Öhberg et al. (1996). A network is initially trained using the ground-truth followed by the data classification. Neural networks are perfect examples of such algorithms (Veerabhadrappa et al., 2015). Since the raw extracellular data does not possess any ground-truth information, training of a neural network is not possible. Henceforth, Neural-Network based clustering has not been considered in our study. Some of the notable classifiers are: (a) Leader-follower algorithm, (b) Support Vector Machines and (c) Growing Neural Gas.

### 2.2. Criteria for Choosing Cluster Algorithms in the Current Review

With such a wide range of algorithms reported, the selection of a particular algorithm is usually subjective Kaufman and Rousseeuw (2008b). Some of the algorithms widely adopted for the purpose of spike sorting are K-means (Pachitariu et al., 2016; Caro-Martín et al., 2018), GMM (Souza et al., 2019), SPC (Blatt et al., 1996; Rey et al., 2015; Niediek et al., 2016), Klustakwik (Rossant et al., 2016), and methods based on statistical aggregations such as t-distributions or *chi*^2^ distribution (Harris et al., 2000; Shan et al., 2017). We reviewed 58 algorithms out of which 27 are presented in the report. The requirements for choosing algorithms is as follows:

- An algorithm is introduced to specifically overcome drawbacks or, was proposed as a better alternative approach. For example (a) Chameleon was introduced to overcome the drawbacks of CURE and ROCK; which ignored to address interconnectivity and close relation between pairs of similar clusters Karypis et al. (1999), (b) EM based algorithm was introduced as an alternative to K-means, and FCM (Shoham et al., 2003), (c) Wave_clus employing SPC was introduced as an alternative to handle outlier insensitivity of K-means (Blatt et al., 1996), and (d) Affinity Propagation (AF_Prop) was introduced as an alternative to K-means and hierarchical clustering algorithms (Frey and Dueck, 2007). Under the above conditions, all the algorithms were considered to understand their differences and advantages to spike sorting.
- Similarly, semantic edge weighting concepts of graphs offered specific advantages in classifying protein-protein interactions (Price et al., 2013). The graph-based algorithms clustering in quest (clique), DPClus, graph-entropy, CoAch, IPCA, and MCODE were selected (Price et al., 2013) to assess their performance in clustering extracellular action potential features.
- Algorithms exclusively employed in spike sorting such as Mean-shift, ISO-SPLIT, OPTICS, EMGMM, VBGMM, SPC, and Klustakwik (details in section 2.1).
- When variants of clustering algorithms such as PAM, CLARA, CLARANS which share relation with K-means and K-medoids, the former have not been considered (Kaufman and Rousseeuw, 2008b).
- Kernel variants of clustering algorithms (K-means, FCM) are not considered because, it is already established that in a linear distribution of features, the resulting outcomes between their convention versions (Kim et al., 2005) is not significantly different. Moreover, since our study employs static parameters, i.e., a fixed number of partitions and feature vectors (details in section 3.2) the necessity of kernel version becomes redundant (Girolami, 2002; Kim et al., 2005). It is also reported that on average kernel-based clustering algorithms can provide only 15% better performance than their conventional versions; for detailed evaluation results and data specifics, we advise the readers to refer to Kim et al. (2005).
- In a strong argument, the article (Ankerst et al., 1999) introduces OPTICS as a better alternative to algorithms such as BANG, CURE, K-means, K-medoids, PAM, CLARA, CLARANS, DBSCAN, density-based clustering (DenClue), clique, BIRCH, and WaveCluster covering up most families of clustering. Hence, in the current study one from each family is selected; CURE, DBSCAN, BIRCH (details of families is discussed in section 2.1 and reason for K-means and K-medoids already stated above) to compare their performance against OPTICS.
- Generic conditions such as (a) publicly accessible (b) application in biomedical signal processing (c) application in spike sorting (d) at least one algorithm possibly from a family (e) readily available in its novelty version and, (f) application of the algorithm is non-trivial. Figure 2 shows popular clustering algorithms which are considered in the report and their immediate relatives, which are not considered but may have a similar impact.

## 3. Materials and Methods

### 3.1. Data Sets

Our study employs three sets of data (two synthetic and a real data set) with ground-truth (Quiroga, 2009). The data sets are chosen such that it facilitates the analyses and evaluating the capacity of a clustering algorithm to distinguish between spike shapes. Hence from Table 1 the data set with *Low* similarity indicates that the spike shapes were relatively easier to distinguish compared to *High* similarity. Further, a real data set represents an example of the spike shape similarity that could be expected in real recordings, compensating any disparity that may exist in synthetic data.

**Table 1:** Details of labels used in the report for referring to a data set and its corresponding feature-set.

**Labels**	Data set from repository (*.mat) (Quiroga et al., 2004)	**Similarity between spike shapes**	**Number of spikes M**	**Number of spike classes**
Eks	C_Easy1_noise_01	Low	3,522	3
Epca	C_Easy1_noise_01		3,522	3
Dks	C_Difficult1_noise_01	High	3,448	3
Dpca	C_Difficult1_noise_01		3,448	3
Uks	time_CSC4	Original	9,193	4
Upca	time_CSC5		9,193	4

Synthetic data sets represented voltage recordings from a single channel of an extracellular recording. During the synthesis of each data set, three different spike shapes were randomly chosen from a pool of 594 spike waveforms acquired from the neocortex and basal ganglia (Quiroga, 2009). We initially band-pass filtered the data sets to remove any field potential and noise, as shown in Figure 1A. Considering the main focus of the study, we ignore the initial spike detection process, thereby, readily available spike times from the ground-truth were used to extract spike waveforms. For each spike time, 20 samples to the left and 44 samples to the right were extracted from filtered voltage to form a spike waveform. The peaks of all the waveforms were aligned at 20th sample constructing a pool of waveforms, as shown in Figure 1C.

Real data set is a collection of waveforms recorded from the temporal lobe of an epileptic patient (Quiroga, 2019) which comprised of four different classes of spike shapes. No additional processing was necessary as the spike waveforms were already extracted and ready for analysis.

The extracted spike waveforms are then processed using PCA and wavelets decomposition to represent raw data into widely used feature vectors. The process undertaken to create features vectors is as follows:

### 3.2. Feature Vector Estimation

#### 3.2.1. PCA Features

Our study adopts PCA feature extraction process similar to the technique documented by Harris et al. (2000). Pooled spike waveforms from each data set were subjected to PCA, and three components with the largest variance (covering 95%) were extracted. Each waveform was then projected on to each of the extracted components constructing a three-dimensional feature-set *F*.

#### 3.2.2. Wavelet Features

The wavelet features were estimated by following the process mentioned by Quiroga et al. (2004). The waveform set was initially decomposed using Haar wavelets and employing discrete wavelet decomposition up to five levels. The wavelets are then subjected to Kolmogrov–Smirnoff test to examine their normality. Ten features with the highest deviation from normal were extracted, constructing a 10-dimensional feature-set *F*.

### 3.3. Graph Generation

The graph-based clustering algorithms require a graph as input with nodes and weights. In our study, all the feature-sets are subjected to KNN algorithm. A KNN tree is constructed assuming the number of nearest neighbors *K*, in the tree to be 11. The input graph is a text file (^*^.txt) comprising of a list of all nearest neighbors such that two data points represented nodes and their relevant Euclidean distance as weights.

### 3.4. Clusters Estimation

The clustering algorithms discussed in the report tend to divide each feature-set *F* into *k* clusters. The result of a clustering algorithm $\vec{C}$ is a vector comprised of integer labels representing a spike class. A supervised clustering procedure *S* requires the users to specify the number of possible *n* partitions as described in Equation (1). The *n* in Equation (1) was selected as 3 and 4 for synthetic and real data set respectively. For a supervised clustering algorithm, clusters $\vec{C}$ were estimated using Equation (1). For an unsupervised clustering algorithm *U*, the clusters $\vec{C}$ were estimated using Equation (2). Euclidean distance was used as a standard method to evaluate the separation between points. Additional information on setting parameters specific to an algorithm is mentioned in the supplementary resource (Appendix C).

(1) $$\begin{array}{r} \vec{C}=S\left(F,n\right) \end{array}$$

(2) $$\begin{array}{r} \vec{C}=U\left(F\right) \end{array}$$

## 4. Clustering Performance Quantification

Quantification of clustering is critical to explore the usefulness and compatibility of these algorithms for spike sorting and analysis. Performance quantification also helps to understand which feature-sets lead to better clustering accuracy for any algorithm. In the present study, quantification of clustering is performed using clustering accuracy as well as selected internal and external validation indices. The confusion matrix is adopted to observe the overall class assignment, and elements of the matrix contribute toward3 accuracy estimation (Amancio et al., 2014). Accuracy derived in relation with the ground-truth information measures an algorithms capacity to assign the spikes into its right class appropriately.

In the clustering and classifier world, accuracy is regarded as a reliable method to define quality (Amancio et al., 2014). Nevertheless, to compute accuracy and confusion matrix, ground-truth information is essential. Here, we first establish confusion matrix which accounts for each label *l* in *ground-truth*. For any feature-set, if $\vec{C}$ represents the outcome of a clustering procedure then, the ground-truth label *l* associated with the feature-set is matched with label *c* in $\vec{C}$. The combination of *l* and *c* with maximum matches represents the best match. This procedure will treat *l* to be equivalent to *c*, forcing to occupy the diagonal of the confusion matrix. If label *l* also matches with a different label in $\vec{C}$, the subsequent match counts will form the remaining array in the confusion matrix. This ensures the condition when *l*≠*c*, their counts will occupy on either side of the confusion matrix, respectively. When there are no additional matches, its relevant element will remain zero. Furthermore, if there are too many clusters formed by a clustering procedure, consequently, this results in additional labels which cannot be fit into the confusion matrix and hence discarded. Thus ensuring that the confusion matrix is formed only for labels in the ground-truth. The confusion matrix diagonal represents counts of all true match, and the remaining elements indicate the level of confusion.

Accuracy is simply the ratio of number of spike samples that were accurately sorted without confusion (i.e., diagonal of confusion matrix) to the total number of spikes *M*. Accuracy of each cluster outcome with respect to its original ground-truth is estimated using Equation (3).

(3) $$\begin{array}{r} Accuracy\;=\;\frac{\sum diag\left(ConfM\right)}{M} \end{array}$$

### 4.1. External Index

The methodology of external indices is similar to accuracy discussed above. If E, represents an external validation criterion, then, the external index *E* of any algorithm about a data set is estimated using Equation (4). The external validation method simply compares the labels generated by an algorithm $\vec{C}$ and data set ground-truth labels *G*. We employed Jaccard and Rand indices in our study.

(4) $$\begin{array}{r} E=\text{E}\left(G,\vec{C}\right) \end{array}$$

Analogous to external indices, **Figure 4** details the indices produced by each algorithm across all feature-sets. The external index produces a *zero* for a worst-case (when certain algorithm fails to produce any cluster) and a *one* for ideal-case (*ground-truth*). Any, corresponding intermediate values relate to the quality of the clustering algorithm. For collective evaluation of algorithms performance across feature-sets, we compute the inverted root mean squared error $R\hat{M}SE$. The root mean squared error (RMS) is initially estimated as shown in Equation 5, and then inverted to obtain $R\hat{M}SE$ (Equation 6).

(5) $$\begin{array}{r} \text{ RMS}{\text{E}}_{\text{a}}=\sqrt{\frac{\sum_{\text{F}}{\left({\text{ g}}_{f}-e_{a,f}\right)}^{2}}{N}} \end{array}$$

(6) $$\begin{array}{r} {R\hat{M}SE}_{a}=1-RMSE_{a} \end{array}$$

where, *N* is total number feature-sets under consideration that is 6, *a* ∈ A, a collection of cluster algorithms, *f* ∈ F, set of features, *g*_*f*_ represents *ground*−*truth* for any feature-set *f* which is equivalent to 1; ideal-case and *e*_*a, f*_ represents external indices for an algorithm *a* and feature-set *f*. A graph of $R\hat{M}SE$ across all algorithms is shown in **Figure 6B**.

### 4.2. Internal Index

Although accuracy measure using confusion matrix adopted in the current study is self-explanatory and reliable, however in the absence of *ground-truth*, becomes irrelevant. In such a scenario, internal indices establish an alternative approach to validate the quality of the clusters estimated. A complete comparison of internal indices is discussed by Zhang et al. (2018). Three internal indices; Ball-Hall (BH) (Ball and Hall, 1965), Trace W (TrW) (Milligan and Cooper, 1985), and Davies-Bouldin (DB) (Davies and Bouldin, 1979) regarded as reliable in extracellular data analysis are selected in the current study. Selection of these three internal indices is based on their consistent behavior through the evaluation. The internal validation method tends to find the barycentre of a cluster by calculating the mean squared distance from the center to clustered points. If *I*, represents an internal validity criterion then, corresponding internal indices, *I* of a clustering outcome $\vec{C}$ and feature *F* was estimated using Equation (7).

(7) $$\begin{array}{r} I=\text{I}\left(F,\vec{C}\right) \end{array}$$

**Figure 3:**
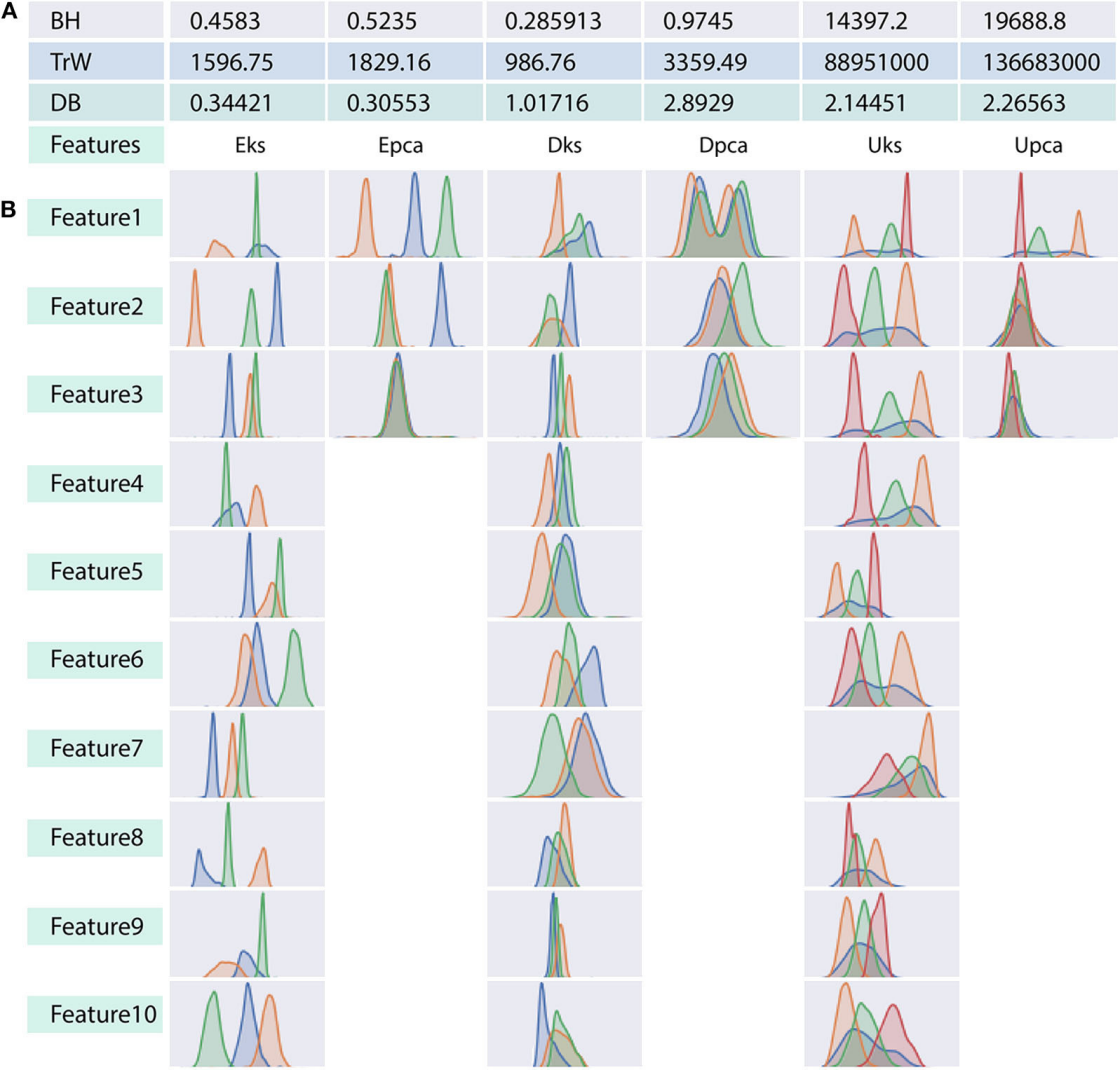
Relation between indices values and distribution plot of clusters in each feature-set. **(A)** Three tabulated rows show indices generated by each of the internal validation criterion considered in the context. Indicies in **(A)** corresponds to the associated ground-truth of each feature-set. **(B)** Distribution of clusters for each dimension in any feature-set. Note that PCA constitutes only three component feature-set as opposed to ten-dimensional feature-set of the wavelet decomposition. We will refer to the overlapped region in **(B)** as ambiguity, which relates to the similarity between spike shapes. This ambiguity distinguishes the performance of clustering algorithms, and it is also harder even for naked eye (human operators) to judge the allocation of feature points to a cluster.

For a better understanding of the ambiguity/confusion associated within a feature-set, we use internal indices as established in Figure 3. From the figure, it is clear that the DB criterion can clearly distinguish the features-sets with a consistent gradient, whereas, the indices of BH and TrW vary depending on the spike count associated with the feature-sets. It should be noted that TrW evaluates indices on an entirely different scale; the values of which are generally ranging in thousands.

Consequently, the range of values produced by each internal validation criteria across the algorithms is very different. For better approximation and uniformity in analysis, we have normalized all the values. Normalized indices compiled by algorithms across all feature-sets are shown in **Figure 5**. Normalization is performed for each pair of internal indices criteria and feature-set. For example, if *I*_*BH, Dks*_ represent internal index values produced by BH criterion for the feature-set *Dks* across all clustering algorithms. Then normalized values of Î_*BH, Dks*_ is estimated using Equation (8). The reference value *r* in Equation (8) is set to index value of *ground-truth*, *I*_*BH, Dks*_(*ground*−*truth*). For collective evaluation, all normalized values are consolidated in **Figure 6A**. The corresponding values of Î will be referenced as normalized internal indices (NII), hereafter.

(8) $$\begin{array}{r} \forall \;i\;in\;I,\;Î=\;1\;-\;\frac{\left|r-i\right|}{\left(max\left(I\right)-min\left(I\right)\right)} \end{array}$$

The interpretation of validation index to select an appropriate cluster may vary from one criterion to another. The cluster results must be selected only while comparing indices values within a feature-set and, bounded by the rules of underlying indices methodology. Approximately five different selection rules have been short-listed, minimum score, maximum score, maximum second derivative, minimum second derivative and maximum difference to left (Zhang et al., 2018). In the current study, DB uses lowest indices scores, BH and TrW uses maximum second difference scores. The following assumption is adopted only as an indicator, to compare indices across data sets, and this should not be used as a method to select cluster result. When comparing the indices values in Figure 3, DB criteria tends to provide more valid information where indices of Epca is low indicating that Epca provides better chances to form appropriate clusters and the same can also be observed in Figure 3B. Likewise, DB indices for Dpca is relatively high, implying that the clusters in the feature-set are not very distinct. Further, BH and TrW also provide equally valid information except that their values are not very much comparable across other feature-sets. This is because the number of data points *M* (spikes count) in the feature-sets impacts the indices score; Table 1 shows *M* for each feature-set. Henceforth, the future discussions will refer to NII Î, **Figure 5**.

## 5. Evaluation and Discussion

**Figure 4:**
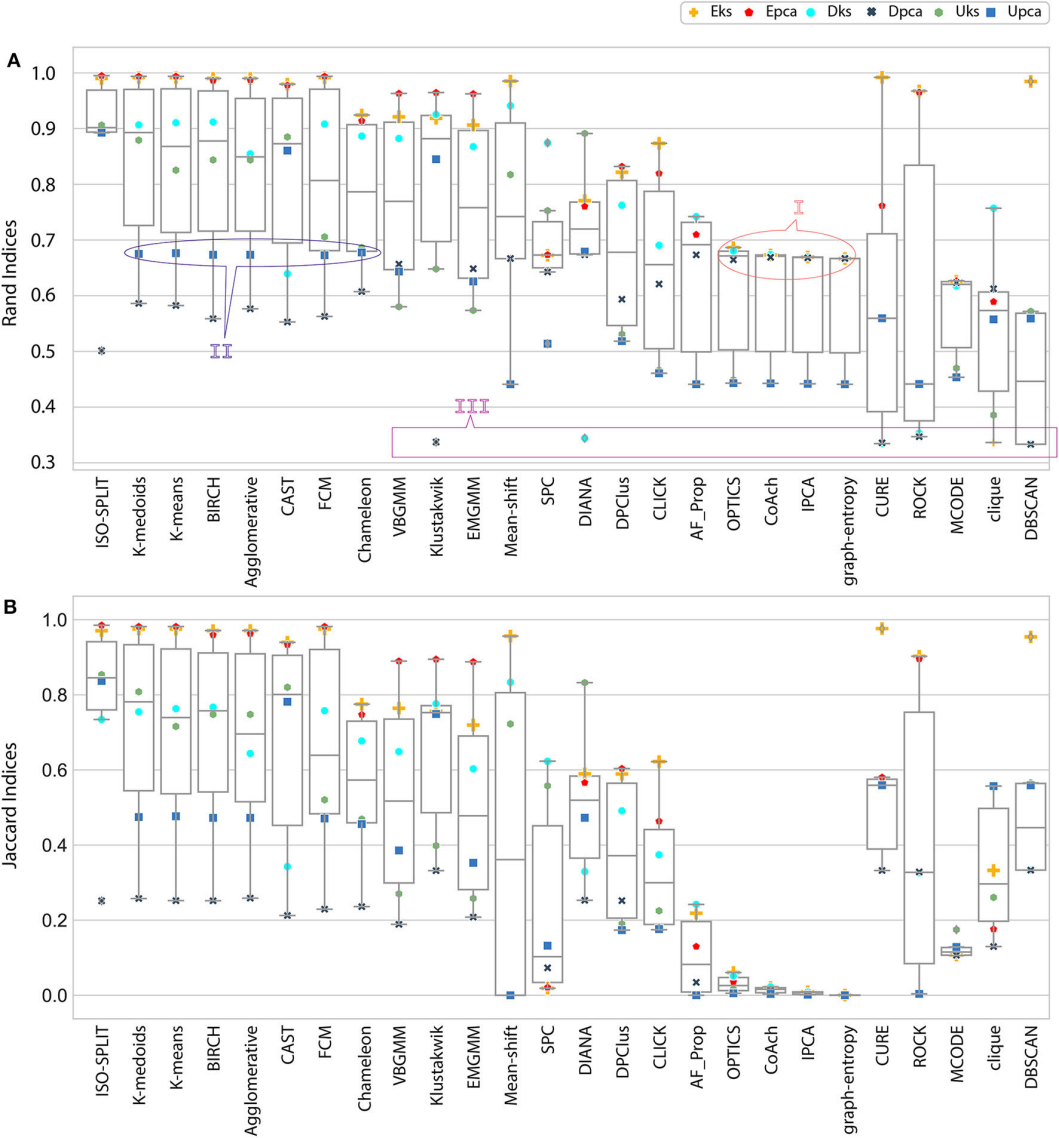
Result of external indices. **(A)** Rand indices, annotations (I, II) refers to a case of confusion whether to be categorized as either good performance or bad performance and annotation III refers to another case where only one cluster was generated (section 5 discusses all annotated cases in detail). **(B)** Jaccard indices. Index value of *zero* indicates no cluster was produced.

**Figure 5:**
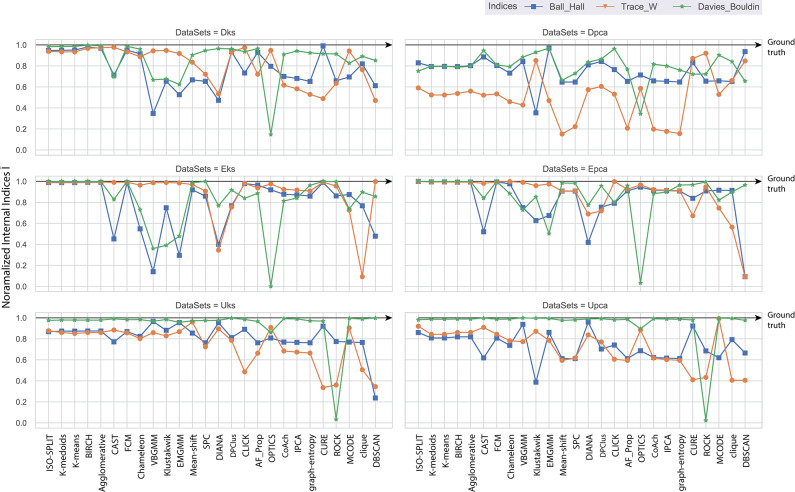
Each plot contains normalized values of three internal indices for each of six feature-set across all algorithms. Results of each internal indices criteria *I* were normalized Î with reference to indices of *ground-truth* labels using Equation (8). The normalization rule is such that a score of *one* indicates *ground-truth* and as score moves away from *one* toward *zero* indicating a drop in performance.

The paper presents an unbiased performance evaluation of 26 clustering algorithms (section 2.1 and Figure 2) via four different evaluation techniques (section 4; internal indices (**Figure 5**), external indices (Figure 4), accuracy and confusion matrix (**Figure 7**). R package *ClusterCrit* was employed for estimating the selected internal and external cluster indices Desgraupes (2014). Data employed in the evaluation provided enough evidence (*ground-truth*) to ensure a fair process was followed. Three sets of data were employed, and two feature-sets from each data were extracted using PCA and wavelet decomposition. For ease of comparison between external and internal indices, the results (range of values) of all three internal indices were normalized to be between *zero* for the worst-case and *one* for the ideal-case using Equation 8. The future analysis and discussions will refer to NII (Î, Equation 8).

**Figure 6:**
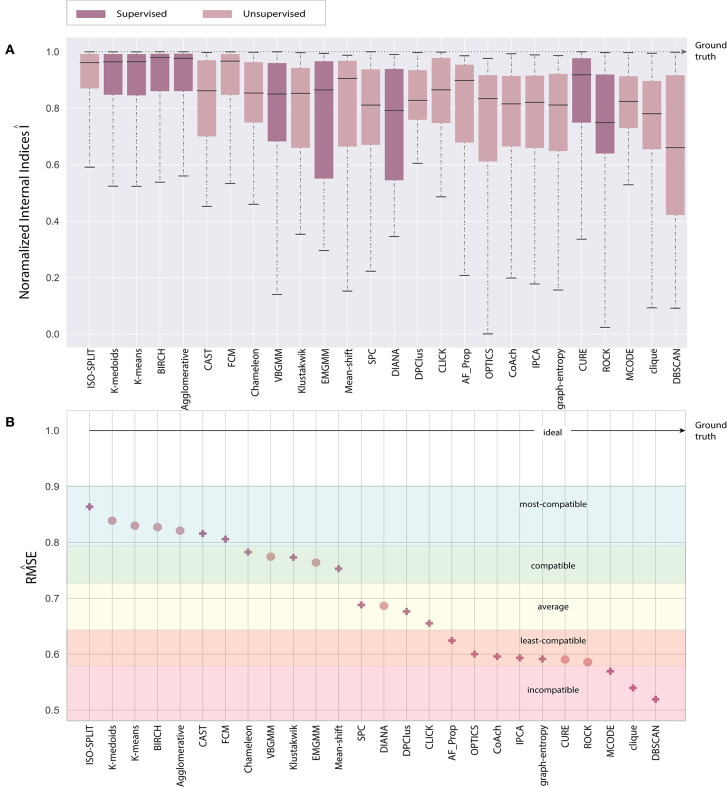
Consolidated results **(A)** normalized internal indices and **(B)** external indices.

Ideally, when comparing evaluations, the expectation is that the indices estimated from results of clustering algorithms should match the indices of *ground-truth*. However, results vary due to ambiguity associated with data and unique processes of a clustering algorithm. From the ambiguity distribution plot in Figure 3, it can be established that feature-sets Eks and Epca possess minimum ambiguity, Dpca represents a difficult degree and remaining feature-sets fall between extreme ends of ambiguity. Attributes of external and internal indices demonstrate the effectiveness of our evaluation process. This process tends to provide an acceptable method for the researchers to incorporate internal indices particularly in the absence of ground-truth effectively. For a generic overview, refer to consolidated results in **Figure 6**.

In the early stages, we explored *feature-set consistency* with the available ground-truth to understand the performances of clustering algorithms. Consistency of algorithms across all 6 feature-sets was evaluated by computing $R\hat{M}SE$ (Equation 6). The algorithms were ranked based on $R\hat{M}SE$ as shown in **Figure 6B**. The ranks were further divided into compatibility category as *ideal, most-compatible, compatible, average, least-compatible* and *non-compatible*. Outcomes of only Rand was considered when computing $R\hat{M}SE$ because Jaccard failed to produce results for more than two algorithms. It is believed that incorporating Rand and Jaccard to estimate $R\hat{M}SE$ does not affect algorithms in *most-compatible* category however, some rankings swap places in categories *average* and after.

**Figure 7:**
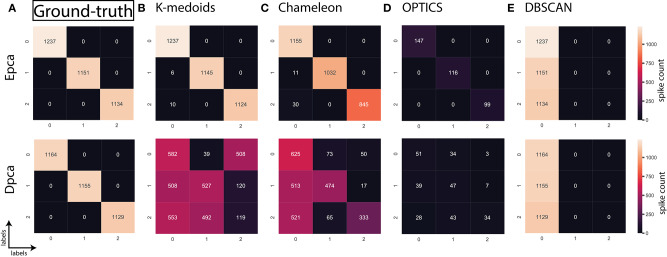
Confusion matrix; relating the performance of an algorithm evaluated using accuracy and false positives. **(A)** Ground-truth: 100% **(B)** K-medoids, Epca: 99.54% and Dpca:35.61%, **(C)** Chameleon, Epca:86.08% and Dpca:41.53%, **(D)** OPTICS, Epca:10.27% and Dpca:3.28%, **(E)** DBSCAN, Epca:33.3% and Dpca:33.3%.

In the absence of ground-truth the NII will aid in better analysis of clusters. The NII of cluster results and ground-truth are compared with each other to observe the effectiveness. For algorithms categorized as *most-compatible* in **Figure 6B**, the corresponding NII outcomes in Figure 5 match close to that of *ground-truth*, and their associated variances in Figure 6A is also minimal. Evidence of this trend can be confirmed through external indices in Figure 4, which complement the high performance of algorithms as illustrated by indices values close to *ground-truth*. Confusion matrix examples of K-medoids and Chameleon from Figures 7B,C further substantiates the previous observations of *most-compatible* algorithms. As with *least-compatible* or *non-compatible* algorithms, the results in Figure 5 show a strong deviation from *ground-truth* and from Figure 4 it is evident that the performance of most algorithms has dropped significantly. As an example, Figures 7D,E confirms the poor performance of DBSCAN and OPTICS. For comparison of clustering algorithm performances, a summary of results is presented in Figure 6 and Table 2 which exhibits a strong agreement between all the evaluation techniques employed in the comparison.

**Table 2:** A summarized version of clustering algorithms performance comparison.

**Algorithms**	**Epca**					**Dpca**				
	**Rand**	**Accuracy**	**DB**	**BH**	**TrW**	**Rand**	**Accuracy**	**DB**	**BH**	**TrW**
Ground-truth	1	100	1	1	1	1	100	1	1	1
K-medoids	0.993	99.545	0.999	0.994	0.995	0.586	35.614	0.797	0.794	0.523
K-means	0.993	99.545	0.999	0.994	0.995	0.582	37.12	0.798	0.795	0.523
Agglomerative	0.987	99.034	0.999	0.993	0.993	0.576	46.635	0.795	0.802	0.559
ISO-SPLIT	0.995	99.63	0.99	0.992	0.99	0.501	35.904	0.751	0.829	0.591
BIRCH	0.986	98.949	0.99	0.99	0.99	0.558	45.852	0.788	0.793	0.537
Chameleon	0.913	86.087	0.883	0.976	0.998	0.607	41.531	0.793	0.731	0.459
CAST	0.977	96.848	0.839	0.52	0.981	0.55	32.221	0.945	0.885	0.521
FCM	0.993	99.545	0.99	0.99	0.99	0.562	37.674	0.81	0.804	0.533
VBGMM	0.963	94.207	0.728	0.750	0.990	0.656	34.106	0.884	0.841	0.427
EMGMM	0.962	94.207	0.503	0.675	0.974	0.648	37.035	0.96	0.96	0.470
SPC	0.673	11.64	0.982	0.907	0.909	0.642	16.502	0.728	0.646	0.222
Mean-shift	0.666	0.454	0.984	0.907	0.908	0.666	0.087	0.663	0.646	0.152
DPClus	0.832	85.178	0.956	0.754	0.719	0.593	49.70	0.86	0.84	0.604
Klustakwik	0.964	94.633	0.853	0.627	0.959	0.337	33.671	0.930	0.353	0.851
CLICK	0.819	64.65	0.798	0.792	0.998	0.62	31.235	0.963	0.765	0.531
DIANA	0.76	65.84	0.774	0.418	0.689	0.673	39.9	0.83	0.8	0.574
AF_Prop	0.7	20.13	0.95	0.9	0.924	0.67	8.613	0.767	0.653	0.207
OPTICS	0.678	10.278	0.033	0.945	0.967	0.664	3.828	0.344	0.714	0.586
CoAch	0.672	3.946	0.883	0.915	0.92	0.66	3.248	0.816	0.657	0.198
IPCA	0.669	1.7	0.898	0.912	0.916	0.667	1.711	0.8	0.653	0.177
graph-entropy	0.66	1.277	0.964	0.9	0.911	0.666	0.754	0.761	0.646	0.155
MCODE	0.626	2.356	0.821	0.915	0.746	0.623	1.856	0.903	0.657	0.528
clique	0.589	7.609	0.89	0.914	0.564	0.612	6.728	0.84	0.652	0.661
CURE	0.761	35.29	0.968	0.838	0.673	0.335	0.2	0.721	0.83	0.871
ROCK	0.965	64.082	0.994	0.9	0.948	0.346	0.29	0.723	0.654	0.92
DBSCAN	0.33	33.33	0.966	0.092	0.091	0.333	33.33	0.655	0.936	0.847

*The outcomes for two different data sets representing the best condition Epca and worst condition Dpca are compared in the following table. The performance is evaluated in terms of rand index, accuracy and NII. The result for remaining of the feature-sets is available in supplementary resource (Appendix B). Note: the results of jaccard is dropped in the table because few algorithms did not generate an index value*.

Further, to strengthen the analogy between internal and external validation indices we will refer to *index-consistency*, consistency among internal indices Figure 5. Observing the results from Figures 6B, 5, *most-compatible* algorithms are characterized by stronger *index-consistency*. As the rank of algorithms moves toward *least-compatible*, consistency between their corresponding NII tends to vary, resulting with a drop in *index-consistency* caused by disagreement among internal indices (example: DBSCAN, ROCK and CURE from Figure 6A shows results with maximum variation). Observe from Figure 5 and Table 2, the NII of *most-compatible* algorithms are fairly consistent across all internal indices. For Chameleon, a barely negligible inconsistency can be observed. However, a noticeable difference can be observed with *average* algorithms. Finally, for OPTICS and DBSCAN, the variation of results among NII is high. This also shows that relying on just one validation method may not provide accurate outcomes. As an example, observe that NII outcomes of CLICK, Klustakwik and EMGMM for Dpca match the indices of *ground-truth*, which could be misleading. It is very clear from the Table 2 that less inconsistency in internal indices leads to better accuracy. The accuracy and confusion matrix tend to agree with the above observations.

A summary of the above observation and discussion reveals that when a feature-set exhibits higher levels of ambiguity or when an algorithm fails to deliver a good performance, the scenario will always result in a disagreement between internal indices. This disagreement will result in higher variances, and the phenomenon can be used as an indication of an *incompatible* algorithm.

At this stage, we would like to discuss some of the shortcomings of external indices (Rand) and possible ways of cross-verifying to accept or reject a clustering result appropriately. The external indices criterion fails to rank clustering results appropriately. This is a condition generally observed with unsupervised approaches where a clustering procedure forms many clusters, resulting in many unnecessary labels. This results in unfairly ranking of algorithms, *least-compatible* algorithms may end up being ranked above *most-compatible* algorithms. A concerning phenomenon could be observed in Figure 4A (annotated *I*), the results generated by *least-compatible* algorithms (CoAch, IPCA, and graph-entropy) for Eks, Epca, Dks, and Dpca have a score of 0.66. A similar score can also be observed from the results generated by *most-compatible* algorithms (K-medoids, K-means, Agglomerative, BIRCH, Chameleon, and FCM) for Upca, and by *compatible* algorithms (EMGMM, Mean-shift and SPC) for Dpca respectively (Figure 4A annotated *II*). The score of 0.66 is considered fairly good in many scenarios, which in this case, the results of *I* could be misleading. Furthermore, observe the confusion matrix of Dpca for different algorithms and their related indices values (Figure 7). Comparing *I* and *II*, it is clear that performances of Chameleon and K-medoids are ranked lower than that of OPTICS where, in reality, Chameleon and K-medoids provide a reliable and acceptable result. It would be practically impossible for a human operator to resolve the inconsistency of OPTICS cluster results. Jaccard however, either fails to generate result in this phenomenon or generates acceptable low indices score. Internal indices scores would be appropriate to approach the phenomenon. Irrespective of ambiguity levels associated with a feature-set, the *index-consistency* is a good indicator of performance. Additionally, internal variances among NII for *least-compatible* algorithms is higher compared to those of a *most-compatible*. The phenomenon can be better observed by comparing NII results for Epca and Dpca (Figure 5). The *index-consistency* among NII for *least-compatible* algorithm is not evident in highly ambiguous feature-set Dpca. On the other hand, *index-consistency* among NII is evident for *most-compatible* as can be observed for lower ambiguous feature-set Epca.

Another shortcoming of indices is when a clustering algorithm returns a cluster where all data points have the same label. An example of such criteria could be observed from Figures 7C,D, having rand indices of 0.33 (Figure 4A annotated *III*). For instance, there are *N* possible clusters in the data-points, in the context of this shortcoming, the validation index returns a value 1/*N*. Although internal indices and NII intend to distinguish good and bad algorithms, DBSCAN and ROCK tend to violate this rule. The raw internal indices closely matched *ground-truth*. From the Table 2 it can be observed for DBSCAN and ROCK, some of the NII are close to 1, in other words *index-consistency* failed to recognize the poor performance. Other popular indices such as “calinski-harabasz” (Buccino et al., 2018) previously employed in the absence of ground-truth did not show any improvements. It is of best interest to avoid these two algorithms specifically for spike sorting.

Overall, the consolidated results in Figure 6 demonstrate that supervised algorithms are mostly consistent and reliable, which is reflected in their stronger *index-consistency* characteristic. From the above discussions, results and in terms of *feature-set consistency* it is clear that supervised algorithms performed better despite higher ambiguity levels. Amongst the unsupervised clustering algorithms the performances of Chameleon, FCM and CAST are fairly competitive, and outperformed clustering algorithms specifically tried for spike sorting; SPC, VBGMM, EMGMM, Klustakwik, and OPTICS. The NII outcomes indicate that majority of probabilistic-based clustering algorithms have similar performances. Fuzzy models performed better than Probabilistic models and that the density-based models such as DBSCAN and OPTICS are not suitable for spike sorting.

Further, Shan et al. in their recent work confirmed the fact that when features demonstrate clear isolation K-means or partition-based clustering perform better than a probabilistic or a gaussian-based model (Shan et al., 2017). Our evaluation also revealed that algorithms which belonged to *most-compatible* and the *compatible* categories performed better when spikes are distinguishable (Epca and Eks). However, a serious limitation is when the spikes are not distinguishable (Dpca and Dks), the performance of most algorithms drop, human operators cannot perform a reliable sorting and validation indices may not provide appropriate result. In a review (Lewicki, 1998), Lewicki advises that software-based spike sorting is necessary to avoid biases, improved decision-making and faster processing. The processing time limitations of human operators can be resolved using a software approach. Moreover, the biases and decision making limitations of clustering algorithms can be resolved by cross verifying the clustering results using multiple algorithms. Comparing the results of Dpca and Dks, wavelet decomposition or signal transformation method can extract better features, thereby mitigating limitations. Our current evaluations suggest that partitional clustering would be a better approach for initial estimation. Then, depending on the requirement, the experimenters could opt for human operators to perform manual clustering followed by probabilistic models to improve the quality of sorting.

## 6. Future Direction of Spike Sorting

In the current testbed, the maximum number of spike classes were four but, in a real scenario, classes of waveforms could vary between 1 to approximately 20 per channel (Pedreira et al., 2012). Data sets with such higher complexity are available to download from http://bioweb.me/CPGJNM2012-dataset (Pedreira et al., 2012; Rey et al., 2015).

None of the algorithms discussed above is entirely reliable. It should be carefully considered whether or not these drawbacks have a significant impact on data under analysis. Researchers should employ a cross-verification method to accept or reject the clusters. Because the raw extracellular data has no *ground-truth*, spike sorting process should include multiple internal indices to assess the performance outcomes. As was claimed in the introduction that not all clustering algorithms are suitable for all types of data, but there are some algorithms which are reasonably consistent across a range of feature-sets. For initial estimation, cross-verification phase or where human-intervention based trial and error approach could employ either of K-medoids, K-means and gaussian mixture models. Additionally, for improvised approach, validation indices and confusion matrix could be effectively used to evaluate the choice of a clustering algorithm. In an event where big-data is concerned and expect a minimal human intervention, our evaluation recommends ISO-SPLIT, FCM, Chameleon and CLICK, as reliable options. The result also shows that a majority of algorithms have responded better for feature-set generated using wavelets. On the other hand, PCA has edged an advantage for larger data sets. In summary, it is clear that supervised procedures perform better than unsupervised procedures.

*Klustakwik* and *Kilo-sort* which employ *phy* suit by far has been a revolution through its versatile user interface, very well received amongst the researchers and has been developed as an open-source platform. The software application can automatically process multiple channels; particularly popular with probe type electrode arrays. Its unique graphical user interface combines necessary information to evaluate an experiment in terms of correlogram, time-series data, cluster results, clustered spikes and similarity report. The adjacency matrix and probe information for *in-vivo* type recordings are available. For *in-vitro* type recording the validity of information available form adjacency matrix is still open to debate. The adjacency matrix and probe information are not available for *in-vitro* microelectrode array recordings to be readily applicable in *klusta* suite. It is also not suitable for CMOS type microelectrode arrays where the channel count is high, which could also result in extensive adjacency matrix.

Similar platforms such as *Spike2* (Ortiz-Rosario et al., 2017), *tridesclous* (upgrade over its less successful predecessors *Spike-O-matic* and *Open Electrophy*) and *Spyke* also provide interactive graphical user interface. The main highlight of *tridesclous* is its flexibility in the choice of clustering technique. The users could choose from K-means, GMM, Agglomerative, DBSCAN or OPTICS Pouzat and Garcia. *Spyke* also uses template matching algorithm (Spacek et al., 2009) for clustering which is based on *Klustakwik* clustering algorithm (Blanche et al., 2004).

There is a need for Klusta-suit type platform which also accommodate reliable clustering algorithms, multiple choice of feature selection processes and provide the user with validation indices to best approximate the outcome. CMOS type microelectrode arrays are gaining popularity, and the data acquisition technique may get more sophisticated. Thus a robust processing system with interactive cluster merging and a numerically aided mechanism to guide human operators is needed for future spike sorting algorithms.

## Data Availability Statement

All datasets generated for this study are included in the article/Supplementary Material.

## Author Contributions

All authors listed have made a substantial, direct and intellectual contribution to the work, and approved it for publication.

## Conflict of Interest

The authors declare that the research was conducted in the absence of any commercial or financial relationships that could be construed as a potential conflict of interest.
