# Supplementary material for: Compatibility Evaluation of Clustering Algorithms for Contemporary Extracellular Neural Spike Sorting
Source: Front Syst Neurosci. 2020 Jun 30;14:34. doi: 10.3389/fnsys.2020.00034 (PMC7340107; doi:10.3389/fnsys.2020.00034)
Supplement: Supplementary file 1 [file Data_Sheet_1.zip › frontiers_SupplementaryMaterial.pdf]

frontiers

Supplementary Material

1 APPENDIX A

Table S1. List of Abbreviations

Affinity Propagation (AF_Prop)	Affinity Propagation
Balanced Iterative Reducing and Clustering using Hierarchies (BIRCH)	Balanced Iterative Reducing and Clustering using Hierarchies
Ball-Hall (BH)	Ball-Hall
Cluster Affinity Search Technique (CAST)	Cluster Affinity Search Technique
Cluster Identificaton using Connectivity Kernels (CLICK)	Cluster Identificaton using Connectivity Kernels
clustering in quest (clique)	clustering in quest
Clustering Large Applications (CLARA)	Clustering Large Applications
Clustering Large Applications based on Randomized Search (CLARANS)	Clustering Large Applications based on Randomized Search
clustering using representatives (CURE)	clustering using representatives
core-attachment method clustering (CoAch)	core-attachment method clustering
Davies-Bouldin (DB)	Davies-Bouldin
Density Based Clustering of Applications with Noise (DBSCAN)	Density Based Clustering of Applications with Noise
density-based clustering (DenClue)	density-based clustering
Divisive Analysis (DIANA)	Divisive Analysis
divisive projected clustering (DPClus)	divisive projected clustering
Expectation Maximization (EM)	Expectation Maximization
expectation maximization based gaussian mixture model (EMGMM)	expectation maximization based gaussian mixture model
Fuzzy C-Means (FCM)	Fuzzy C-Means
Gaussian mixture models (GMM)	Gaussian mixture models
influence power based clustering algorithm (IPCA)	influence power based clustering algorithm
K-nearest neighbour (KNN)	K-nearest neighbour
Minimal Spanning Tree (MST)	Minimal Spanning Tree
molecular complex detection (MCODE)	molecular complex detection
normalised internal indices (NII)	normalised internal indices
ordering points to identify clustering structure (OPTICS)	ordering points to identify clustering structure
partitioning around medoids (PAM)	partitioning around medoids
principal component analysis (PCA)	principal component analysis
robust clustering (ROCK)	robust clustering
root mean squared error (RMS)	root mean squared error
superparamagnetic clustering (SPC)	superparamagnetic clustering
Trace W (TrW)TrWTrace W	Trace W
variational bayesian inference gaussian mixture model (VBGMM)	variational bayesian inference gaussian mixture model

2 APPENDIX B

Table S2. Results of all clustering algorithms for feature-sets Eks, Dks, Uks, and Upca

Algorithms	Eks					Dks				
	Rand	Accu-racy	DB	BH	TrW	Rand	Accu-racy	DB	BH	TrW
original	1	100	1	1	1	1	100	1	1	1
K-medoids	0.99	99.37	0.99	0.98	0.99	0.9	92.54	0.98	0.95	0.93
K-means	0.99	99.37	0.99	0.98	0.99	0.91	92.83	0.98	0.95	0.93
Agglomerative	0.99	99.26	0.99	0.99	0.99	0.85	87.90	0.99	0.96	0.97
ISO-SPLIT	0.99	99.26	0.99	0.98	0.99	0.89	91.87	0.98	0.94	0.93
BIRCH	0.99	99.26	0.99	0.99	0.99	0.91	92.89	0.99	0.98	0.96
Chameleon	0.92	87.81	0.73	0.54	0.96	0.88	83.58	0.95	0.91	0.88
CAST	0.97	97.01	0.82	0.45	0.99	0.63	54.69	0.69	0.71	0.97
FCM	0.99	99.37	0.99	0.98	0.99	0.90	92.63	0.98	0.95	0.93
VBGMM	0.92	80.29	0.36	0.14	0.98	0.88	73.40	0.66	0.34	0.94
EMGMM	0.90	76.14	0.47	0.29	0.98	0.86	65.31	0.62	0.52	0.91
SPC	0.67	8.14	0.99	0.85	0.90	0.87	78.94	0.94	0.65	0.72
Mean-shift	0.98	97.98	0.98	0.91	0.97	0.94	94.08	0.9	0.66	0.83
DPClus	0.82	82.73	0.91	0.76	0.75	0.76	76.07	0.96	0.93	0.93
Klustakwik	0.91	79.64	0.39	0.74	0.98	0.92	82.45	0.67	0.65	0.94
CLICK	0.87	75.89	0.83	0.97	0.98	0.69	65.4	0.93	0.73	0.97
DIANA	0.77	67.37	0.76	0.4	0.34	0.34	33.7	0.96	0.47	0.53
AF_Prop	0.73	27.93	0.88	0.96	0.93	0.74	33.72	0.96	0.92	0.72
OPTICS	0.68	17.17	0	0.91	0.97	0.68	12.38	0.14	0.79	0.94
CoAch	0.67	4.25	0.81	0.87	0.92	0.67	4.78	0.9	0.69	0.61
IPCA	0.66	1.22	0.84	0.87	0.91	0.66	0.58	0.94	0.68	0.58
graph-entropy	0.66	1.24	0.96	0.86	0.9	0.66	0.78	0.92	0.65	0.52
MCODE	0.62	3.35	0.73	0.87	0.72	0.61	3.3	0.82	0.69	0.94
clique	0.33	0.22	0.89	0.76	0.09	0.75	64.29	0.89	0.81	0.76
CURE	0.99	66.83	0.99	0.98	0.99	0.33	0.11	0.91	0.99	0.48
ROCK	0.96	64.48	0.99	0.86	0.95	0.35	0.2	0.91	0.65	0.63
DBSCAN	0.98	97.87	0.85	0.47	0.99	0.33	33.3	0.85	0.61	0.47

Algorithms	Uks					Upca				
	Rand	Accu-racy	DB	BH	TrW	Rand	Accu-racy	DB	BH	TrW
original	1	100	1	1	1	1	100	1	1	1
K-medoids	0.87	80.39	0.97	0.87	0.86	0.67	56.95	0.98	0.8	0.84
K-means	0.82	78.86	0.97	0.87	0.85	0.67	57.61	0.98	0.8	0.84
Agglomerative	0.84	80.88	0.97	0.87	0.85	0.67	55.45	0.98	0.81	0.86
ISO-SPLIT	0.9	88.33	0.97	0.86	0.87	0.89	87.85	0.98	0.86	0.91
BIRCH	0.84	80.88	0.97	0.87	0.85	0.67	55.45	0.98	0.81	0.86
Chameleon	0.68	64.02	0.98	0.82	0.8	0.67	62.39	0.98	0.73	0.78
CAST	0.88	86.29	0.99	0.77	0.88	0.86	84.31	0.99	0.61	0.9
FCM	0.70	63.96	0.98	0.86	0.85	0.67	55.49	0.98	0.80	0.84
VBGMM	0.58	39.59	0.96	0.96	0.85	0.64	52.74	0.99	0.93	0.77
EMGMM	0.57	34.04	0.95	0.95	0.86	0.62	46.22	0.99	0.86	0.78
SPC	0.75	62.41	0.97	0.76	0.72	0.51	27.33	0.98	0.61	0.61
Mean-shift	0.81	82.02	0.97	0.85	0.96	0.44	0.01	0.97	0.61	0.59
DPClus	0.53	40.44	0.99	0.81	0.78	0.51	24.76	0.99	0.7	0.77
Klustakwik	0.64	50.39	0.98	0.88	0.82	0.84	81.18	0.99	0.38	0.87
CLICK	0.46	27.06	0.98	0.89	0.48	0.46	26.44	0.98	0.74	0.6
DIANA	0.89	87.21	0.97	0.95	0.89	0.67	55.78	0.99	0.95	0.83
AF_Prop	0.44	0.04	0.96	0.76	0.66	0.44	0.2	0.98	0.61	0.59
OPTICS	0.44	4.28	0.85	0.8	0.9	0.44	2.0	0.89	0.68	0.88
CoAch	0.44	1.80	0.99	0.76	0.68	0.44	1.15	0.99	0.62	0.61
IPCA	0.44	0.69	0.98	0.76	0.67	0.44	0.53	0.98	0.61	0.6
graph-entropy	0.44	0.28	0.97	0.76	0.66	0.44	0.27	0.98	0.61	0.59
MCODE	0.46	1.14	0.99	0.76	0.9	0.45	0.98	0.99	0.62	0.99
clique	0.38	3.7	0.98	0.76	0.5	0.55	0.08	0.99	0.79	0.4
CURE	0.55	53.9	0.96	0.92	0.33	0.55	55.7	0.97	0.92	0.41
ROCK	0.44	1.07	0.03	0.77	0.36	0.44	0.52	0.023	0.68	0.43
DBSCAN	0.57	72.19	0.99	0.23	0.34	0.55	33.3	0.97	0.66	0.4

3 APPENDIX C

Table S3: Details of source used in the report

Notation used in report	Algorithm	Source [url]	input parameters	Ability to handle large data sets	Ability to high dimensional data sets	Resilience towards noise	time complexity	Software package (if available)	Citation
clique	Clustering in quest		cliques size	Poor	Good	Moderate	Low $O(n+k^2)$	Python	Price et al. (2013); Palla et al. (2005)
DPClus	Divisive projected clustering		cluster density, cluster property	-	-	-	-	-	Price et al. (2013); Altaf-Ul-Amin et al. (2006)
graph-entropy	graph-entropy		-		-	-	-	-	Price et al. (2013); Kenley and Cho (2011)
CoAch	core-attachment method clustering		Density Threshold, Affinity Threshold, Closeness threshold	-	-	-	-	-	Price et al. (2013); Wu et al. (2009)
IPCA	Influence power based clustering algorithm	https://github.com/trueprice/python-graph-clustering.git	Minimum % of adjacent cluster nodes, Shortest path	-	-	-	-	-	Price et al. (2013); Li et al. (2008)
MCODE	molecular complex detection		weight threshold	-	-	-	-	-	Price et al. (2013); Bader and Hogue (2003)
Chameleon	Chameleon Clustering	https://github.com/Moonpuck/chameleon_cluster<br>https://github.com/giovannipcarvalho/PyCHAMELEON		Poor	Poor	High	High $O(n^2)$	Python	Karypis et al. (1999)<br>Karypis et al. (1999)

Notation used in report	Algorithm	Source [url]	input parameters	Ability to handle large data sets	Ability to high dimensional data sets	Resilience towards noise	time complexity	Software package (if available)	Citation
CURE	Clustering Using Representatives		min cluster size	Good	Good	High	Low $O(s^2 * logs)$	python package: pyclus	Novikov (2019); Guha et al. (1998)
DIANA	Divisive Hierarchial Clustering: Implemenation name Divisive Analysis or Di-Ana)	https://stat.ethz.ch/R-manual/R-devel/library/cluster/html/diana.html	min cluster size	-	-	-	-	R Package: cluster	Dia (2008)
Klustakwik	Klustakwik	https://pypi.org/project/klustakwik2/	-	-	-	-	-	Python: Klusta2 suite	Rossant et al. (2016); Kadir et al. (2014)
CAST	Cluster Affinity Search Technique	http://mev.tm4.org	threshold	-	-	-	-	TIGR software	Ben-Dor et al. (1999); Howe et al. (2010)
CLICK		http://www.cs.tau.ac.il/~rshamir/expander/expander.html	homogeneity value	-	-	-	-	Expander software	Sharan and Shamir (2000); Shamir et al. (2005)
Wave_clus(SPC	Wave_clus tool uses Super-paramagnetic clustering method	https://www2.le.ac.uk/departments/engineering/research/bioengineering/neuroengineering-lab/spike-sorting	Temperature range(min, max, step-size), Swendsen-wang cycles, Nearest Neighbours, clusters(min and max)	-	-	-	-	wave_clus matlab implementation suite	Quiroga et al. (2004)
spectral_clus	Spectral Clustering		graph, min cluster size	Poor	Good	High	High (eigenvector)	python package: scikit-learn, SpectralClustering	Pedregosa et al. (2011); Shi and Malik (2000)

Notation used in report	Algorithm	Source [url]	input parameters	Ability to handle large data sets	Ability to high dimensional data sets	Resilience towards noise	time complexity	Software package (if available)	Citation
K-means	K-means		graph, min cluster size	Good	Poor	High	High (kernel)	python package: scikit-learn, KMeans	Pedregosa et al. (2011); Salganicoff et al. (1988); Caro-Martín et al. (2018)
Mean-shift	Mean-shift		bandwidth	Poor	Poor	High	High (kernel)	python package: scikit-learn, MeanShift	Pedregosa et al. (2011)
BIRCH	Balanced Iterative Reducing and Clustering using Hierarchies	https://scikit-learn.org/stable/modules/clustering.html#clustering	min cluster size	-	-	-	-	python package: scikit-learn, Birch	Pedregosa et al. (2011); Zhang et al. (1996)
Agglomerative	Agglomerative clustering, using single linkage		min cluster size	Poor	Poor	Low	High (iterations)	python package: scikit-learn, AgglomerativeClustering	Pedregosa et al. (2011)
DBSCAN	Density Based Spatial Clustering of Application with Noise		epsilon	Good	Poor	High	Moderate $O(n * logn)$	python package: scikit-learn, DBSCAN	Pedregosa et al. (2011)
OPTICS	Ordering Points to Identify Clustering Structure		min cluster size, min samples	Good	Poor	High	Moderate $O(n * logn)$	python package: scikit-learn, OPTICS	Pedregosa et al. (2011)
AF_Prop	Affinity Propogation		min cluster size, damping factor	Poor	Poor	High	High $O(n^2 * logn)$	python package: scikit-learn, AffinityPropagation	Pedregosa et al. (2011)
K-medoids	K-medoids	https://pypi.org/project/pyclustering/	min cluster size	Poor	Poor	High	High $O(k(n-k)^2)$	python package: pyclustering	Pedregosa et al. (2011)

Notation used in report	Algorithm	Source [url]	input parameters	Ability to handle large data sets	Ability to high dimensional data sets	Resilience towards noise	time complexity	Software package (if available)	Citation
VBGMM	Variational Bayesian Inference Gaussian Mixture Model	https://au.mathworks.com/matlabcentral/fileexchange/35362-variational-bayesian-inference-for-gaussian-mixture-model	min cluster size	Poor	Poor	High	High $O(n^2 * kt)$	Matlab	Bishop (2006a)
EMGMM	Gaussian Mixture Model using Expectation Maximization	https://au.mathworks.com/matlabcentral/fileexchange/26184-em-algorithm-for-gaussian-mixture-model-em-gmm	min cluster size	Poor	Poor	High	High $O(n^2 * kt)$	Matlab	Bishop (2006b)
FCM	Fuzzy C-Means	https://au.mathworks.com/help/fuzzy/fcm.html	min cluster size	Poor	Poor	low	low $O(n)$	Matlab	Zouridakis and Tam (2000)
ISO-SPLIT	ISO-SPLIT	https://github.com/flatironinstitute/isosplit5	-	-	-	-	-	Matlab	Magland and Barnett (2015); Chung et al. (2017)
PAM	Partitioning Around Medoids	https://www.datanovia.com/en/lessons/k-medoids-in-r-algorithm-and-practical-examples/	min cluster size	Poor	Poor	High	High $O(k^3 * n^2)$	R Package	Xu and Tian (2015)
CLARANS	Clustering Large Applications based on RANdomized Search	https://pypi.org/project/pyclustering/	min cluster size, maximum neighbours	Good	Poor	High	High $O(n^2)$	python package: pyclustering	Xu and Tian (2015)
CLARA	Clustering Large Applications	https://www.datanovia.com/en/lessons/clara-in-r-clustering-large-applications/	min cluster size	Good	Poor	High	Moderate $O(ks^2 + k(n-k))$	R Package	Xu and Tian (2015)
FCS	fuzzy c-shells	http://ugrad.stat.ubc.ca/R/library/e1071/html/cshell.html	min cluster size, iterations	Poor	Poor	Low	High (kernel)	R Package	Xu and Tian (2015)
DBCLASD	Distribution-Based Clustering of LArge Spatial Databases	https://github.com/spalaciob/py-dbclasd/blob/master/dbclasd.py	-	Good	Good	Low	Moderate $O(n * logn)$	Pyhton Package	Xu and Tian (2015)
MCLUST	model-based clustering	https://cran.r-project.org/web/packages/mclust/vignettes/mclust.html	-	-	-	-	-	R package	Xu and Tian (2015)

Notation used in report	Algorithm	Source [url]	input parameters	Ability to handle large data sets	Ability to high dimensional data sets	Resilience towards noise	time complexity	Software package (if available)	Citation
DENCLUE	Density based clustering	`https://haifengl.github.io/smile/api/java/smile/clustering/DENCLUE.html`	-	-	-	-	-	Pyhton and Java	Xu and Tian (2015)
MST	minimum spanning tree	`https://cran.r-project.org/web/packages/mstknnclust/vignettes/guide.html`	min cluster size	Good	Poor	Low	Moderate $O(e * logv)$	R package	Xu and Tian (2015)
LF	Leader Follower Clustering	`https://cran.r-project.org/web/packages/leaderCluster/index.html`		Poor	Poor	Low	High (iterations)	R Package	Xu and Tian (2015)
SOM	Self Organising Map	`https://au.mathworks.com/help/deeplearning/gs/cluster-data-with-a-self-organizing-map.html`	Neural Network training	Poor	Good	High	High (layer)	Matlab Tool Kit	Xu and Tian (2015)
SVM	Support Vector Machine Clustering	`https://scikit-learn.org/stable/modules/svm.html`		-	-	-	-	Python Package	Xu and Tian (2015)
GNG	Growing Neural Gas	`http://neupy.com/2018/03/26/making_art_with_growing_neural_gas.html`		Good	Poor	Low	Moderate $(type + layer)$	Python Package	Xu and Tian (2015)
ART	adaptive resonance theory	`https://github.com/chen0040/java-adaptive-resonance-theory`	-	Good	Good	Low	Low $O(n)$	Java Package	Xu and Tian (2015)
STING	STatistical Information Grid		-	-	-	-	-	Matlab	Xu and Tian (2015)
Wavecluster	WaveCluster Algorithm		-	-	-	-	-	Pseudu Code	Xu and Tian (2015)
FC	functional clustering	`https://rdrr.io/cran/fdapace/man/FClust.html`	time of sample origin, min cluster size	Good	Good	High	Low$(O(n))$	R package	Xu and Tian (2015)
COBWEB	COBWEB Clustering	-	-	Good	Poor	Moderate	Low (distribution)	Matlab	
GMM	Gaussian mixture model	-	-	Poor	Poor	High	High $O(n^2 * kt)$	Matlab	Xu and Tian (2015)
Kernel Kmeans	kernel k-means Clustering	`https://gist.github.com/mblondel/6230787`	min cluster size, iterations, kernel type	Poor	Poor	Low	High (kernel)	Python Package	Xu and Tian (2015)

Notation used in report	Algorithm	Source [url]	input parameters	Ability to handle large data sets	Ability to high dimensional data sets	Resilience towards noise	time complexity	Software package (if available)	Citation
Kernel SOM	Kernel Self-Organizing Map Clustering Algorithm	https://rdrr.io/rforge/yasomi/man/batchsom.kernelmatrix.html	min cluster size, iterations, kernel type	Poor	Poor	Low	High (kernel)	R package	Xu and Tian (2015)
Kernel FCM	kernel fuzzy c-means	-	-	Poor	Poor	Low	High (kernel)	Matlab	Xu and Tian (2015)
SVC	Support Vector Clustering	https://rdrr.io/cran/SwarmSVM/man/clusterSVM.html	-	-	-	-	-	R Package	Xu and Tian (2015)
MMC	Modulated Modularity Clustering	https://rdrr.io/bioc/scran/man/clusterModularity.html	graph, min cluster size	Poor	Poor	Low	High (kernel)	R package	Xu and Tian (2015)
MKC	Multiple kernel clustering	https://rdrr.io/github/SeojinBang/MKKC/man/mkkc.html	graph, min cluster size	Poor	Poor	Low	High (kernel)	R package	Xu and Tian (2015)
ACO_based (LF)	Ant Colony Optimization	https://rdrr.io/cran/ShortForm/man/antcolony.lavaan.html	ants, evaporation, steps, max runs	Poor	Poor	Low	High (iterations)	R Package	Xu and Tian (2015)
PSO_based	Particle swarm optimization	https://au.mathworks.com/matlabcentral/fileexchange/7506-particle-swarm-optimization-toolbox	lower bounds, upper bounds, omega, swarm size, max iteration	Poor	Poor	Low	High (iterations)	Matlab	Xu and Tian (2015)
SFLA_based	shuffled frog-leaping algorithm	https://rdrr.io/cran/metaheuristicOpt/man/SFL.html	cost function, max iteration, number of population to be detected	Poor	Poor	Low	High (iterations)	R package	Xu and Tian (2015)
ABC_based	Artificial Bee Colony	https://rdrr.io/cran/ABCoptim/man/ABCoptim-package.html	-	Poor	Poor	Low	High (iterations)	R package	Xu and Tian (2015)
QC	Quantum clustering	https://github.com/sliorde/quantum-clustering	standard deviation from gaussians	Poor	Poor	High	High (schrodinger equation + iteration)	Python, Matlab, C	Xu and Tian (2015)
DQC	Dynamic Quantum clustering	https://github.com/peterwittek/dqc-gpu		Poor	Poor	High	Moderate (schrodinger equation)	C++	Xu and Tian (2015)
HDBSCAN	Hierarchical Density-Based Spatial Clustering of Applications with Noise	https://hdbscan.readthedocs.io/en/latest/how_hdbscan_works.html	alpha	Good	Poor	High	Middle $O(n * logn)$	Python	Xu and Tian (2015)
